# Comprehensive polymerase chain reaction assay for detection of pathogenic DNA in lymphoproliferative disorders of the ocular adnexa

**DOI:** 10.1038/srep36621

**Published:** 2016-11-10

**Authors:** Yoshihiko Usui, Narsing A. Rao, Hiroshi Takase, Kinya Tsubota, Kazuhiko Umazume, Daniel Diaz-Aguilar, Takeshi Kezuka, Manabu Mochizuki, Hiroshi Goto, Sunao Sugita

**Affiliations:** 1 Department of Ophthalmology, Tokyo Medical University, Tokyo, Japan; 2 Department of Ophthalmology, University of Southern California, USA; 3 Department of Ophthalmology & Visual Science, Tokyo Medical and Dental University, Tokyo, Japan; 4 David Geffen School of Medicine, University of California, 10833 Le Conte Ave, Los Angeles, CA 90095; 5 Miyata Eye Hospital, Miyakonojo, Japan; 6 Laboratory for Retinal Regeneration, RIKEN Center for Developmental Biology, Kobe, Japan; 7 Department of Ophthalmology, Kobe City Medical Center General Hospital, Kobe, Japan

## Abstract

Infectious agents have been identified as a major cause of specific types of human cancers worldwide. Several microorganisms have been identified as potential aggravators of ocular adnexal neoplasms; however, given the rarity of these neoplasms, large epidemiological studies are difficult to coordinate. This study aimed to conduct an exhaustive search for pathogenic DNA in lymphoproliferative disorders (LPD) of the ocular adnexa in a total of 70 patients who were diagnosed with LPD of the ocular adnexa between 2008 and 2013. Specimens were screened for bacterial, viral, fungal, and parasitic DNA by multiplex polymerase chain reaction (PCR) and quantitative real-time PCR. Among cases of conjunctival mucosa-associated lymphoid tissue lymphoma, human herpes virus (HHV)-6, HHV-7, chlamydia, Epstein-Barr virus (EBV) and bacterial 16S ribosomal DNA were detected. In cases of IgG4-related ocular disease, similar pathogens were detected but in a larger number of patients. Our PCR assays detected DNAs of various infectious agents in tumor specimens, especially HHV6, HHV7, and EBV, with different positive rates in various types of LPD. Chronic inflammatory stimulation or activation of oncogenes from these infectious agents might be involved in the pathogenesis of LPD of the ocular adnexa.

The link between chronic viral, bacterial, or parasitic stimulation and the development of specific malignancies in humans are well described^1^. Long-standing infection with specific pathogens is implicated in the origin and development of several benign and malignant lymphoproliferative disorders (LPD). Examples of well-studied malignant associations include *Helicobacter pylori* infection in mucosa-associated lymphoid tissue (MALT) lymphoma; hepatitis B virus (HBV) and hepatitis C virus (HCV) in hepatic carcinoma; and Epstein-Barr virus (EBV) in Hodgkin’s and Burkitt’s lymphoma^2^. Malignant transformation of lymphocytes in the context of viral or bacterial infection is a multi-dimensional process. Certain viruses such as EBV and HTLV1 directly infect lymphocytes, inducing lymphoid hyperplasia and malignant transformation over time. However, direct antigen stimulation is not always necessary for the development of lymphoma. For instance, *H. pylori*-induced chronic gastritis progresses to MALT lymphoma via chronic inflammation and indirect stimulation of lymphocytes. These direct and indirect stimulations of lymphocytes through chronic infectious disease may also play a role in the development of less well-studied benign LPDs, such as IgG4-related disease^3^.

LPDs of the ocular adnexa comprise a wide spectrum of disorders from benign lymphoid hyperplasia to monoclonal malignant lymphoma, with a range of incidence and potential origins. In Japan, MALT lymphoma is the most frequent primary malignancy of the ocular adnexa region, whereas IgG4-related ophthalmic disease (IgG4-ROD) and reactive lymphoid hyperplasia (RLH) are the most common benign LPDs of the ocular adnexa^4^,^5^. The etiologies of most LPDs of the ocular adnexa have been widely investigated, but several other disorders including IgG4-ROD require further study. IgG4-ROD is a recently recognized syndrome with clinical features that mimic malignant lymphoma, including local and systemic lymphadenopathy and vascular proliferation, which persist and relapse over time^3^,^5^. Although long-term outcomes of patients with IgG4-ROD are generally favorable, there are reports of increased mortality due to systemic effects including multiple organ involvement and damage^6^.

The natural history and pathogenesis of several LPDs are not completely understood. A potentially promising line of study, however, implicates chronic antigen stimulation as a causative agent in the development of benign or malignant lymphoproliferative processes^7^,^8^,^9^.

Several studies have investigated the possible association between LPDs of the ocular adnexa and infectious agents such as herpes simplex virus (HSV)-1, HSV-2, adenovirus 8, HCV, *Helicobacter pylori*, *Chlamydia psittaci*, *Chlamydia trachomatis*, *Chlamydophila abortus*, and *Chlamydophila pneumoniae*^10^,^11^. The findings suggest that infectious agents are associated with the pathogenesis of LPDs of the ocular adnexa. This hypothesis led us to examine the possible association of infectious agents and LPDs of the ocular adnexa. The multiplex and broad-range polymerase chain reaction (PCR) system has been used clinically for simultaneous detection of many pathogens in a very small sample volume of ocular fluids and the diagnosis of infectious uveitis^12^. Using this method, we investigated the presence of 23 pathogenic DNA in tissue specimens of ocular adnexa with various LPDs.

The aim of the present study was to comprehensively examine various infectious agents in ocular adnexa with LPDs using the multiplex and broad-range PCR systems, which might be useful for detecting unexpected infectious associations and elucidating the origins of LPDs of the ocular adnexa.

## Results

### Demographic characteristics and identification of infectious agents in lymphoproliferative disorders of the ocular adnexa

**Figure 1:**
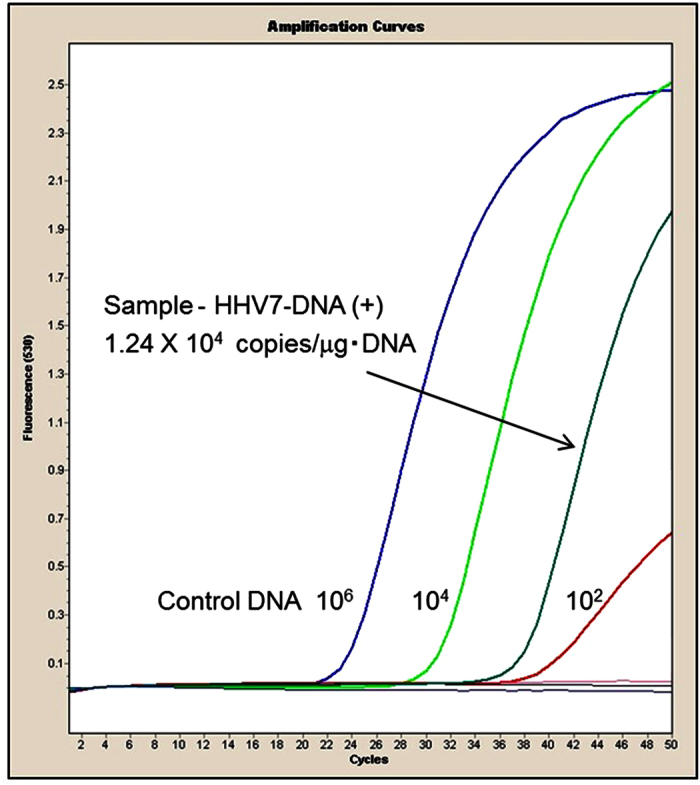
Representative real-time PCR results of a tumor sample obtained from a case of IgG4-related ophthalmic disease. The sample is positive for HHV7-DNA, with 1.24 × 10^6^ copies/μg DNA. Control DNA: standard HHV7-DNA (10^6^, 10^4^, 10^2^ copies).

The patients studied showed no significant difference in age or gender distribution, while patients with conjunctival MALT lymphoma, orbital MALT lymphoma or orbital DLBCL demonstrated IgH gene rearrangement. PCR results of a sample obtained from a representative case are shown in Fig. 1. The results of biopsied specimens in each LPD of the ocular adnexa, in which our comprehensive PCR system showed positivity, are presented in Table 1. Orbital MALT lymphoma tissues revealed a very rate of positivity for infectious agents, with only 1/15 patients demonstrating an HHV-6 infection from the collected biopsy. However, conjunctival MALT lymphoma demonstrated a much higher, but non-specific association with invasive pathogens including EBV, HHV-6, HHV-7 chlamydia and non-specific bacteria. Orbital DLBCL demonstrated a moderate association with EBV presence in 28.6% of all cases. However, IgG4-ROD showed a much stronger association with EBV (31.8%), HHV-6 (22.7%) and HHV-7 (36.4%), and similar values were seen in orbital RLH.

Only one sample of conjunctival MALT lymphoma tested positive for chlamydia 23S (1/19; 6.39 × 10^1^ copies/μg DNA). Our multiplex real-time PCR analyses identified 29 PCR-positive patients. HSV-1, HSV-2, VZV, CMV, HHV-8, BK virus, JC virus, HTLV-1, Coxsackie virus, enterovirus, influenza, *Bartonella*, chlamydia, *Acanthamoeba*, toxocara, toxoplasma, and fungal 18S/28S were not detected in any of the samples. No positivity was found for these microorganisms in peripheral blood mononuclear cells obtained from these patients or in conjunctiva obtained from 23 controls.

### Prevalence of infectious pathogen and presence of multiple co-infections in biopsied specimens of LPD

Members of the herpes family were most commonly associated with the collected samples of LPD. EBV was the most commonly associated agent with 14 samples tested positive for EBV: conjunctival MALT lymphoma (3/19; mean 3.87 × 10^2^ copies/μg DNA), orbital DLBCL (2/7; mean 2.96 × 10^3^ copies/μg DNA), IgG4-ROD (7/22; mean 3.88 × 10^3^ copies/μg DNA), and orbital RLH (2/7; mean 1.22 × 10^2^ copies/μg DNA). Twelve samples tested positive for HHV-7: conjunctival MALT lymphoma (1/19; 1.36 × 10^2^ copies/μg DNA), IgG4-ROD (8/22; mean 5.03 × 10^3^ copies/μg DNA), and orbital RLH (3/7; mean 1.20 × 10^4^ copies/μg DNA). Nine samples tested positive for HHV-6: conjunctival MALT lymphoma (1/19; 1.61 × 10^2^ copies/μg DNA), orbital MALT lymphoma (1/15; 6.10 × 10^1^ copies/μg DNA), IgG4-ROD (5/22; mean 1.71 × 10^3^ copies/μg DNA), and orbital RLH (2/7; mean 1.46 × 10^3^ copies/μg DNA). Five samples tested positive for bacterial 16S: conjunctival MALT lymphoma (2/19; mean 3.60 × 10^3^ copies/μg DNA), orbital DLBCL (1/7; 4.30 × 10^3^ copies/μg DNA), IgG4-ROD (1/22; 2.54 × 10^2^ copies/μg DNA), and orbital RLH (1/7; 1.02 × 10^3^ copies/μg DNA).

As shown in Table 2, double infections (HHV-6 and EBV: 1 case, HHV-7 and EBV: 1 case) were detected in tumor specimens from 2 of 19 patients with conjunctival MALT lymphoma. Furthermore, double infections (HHV-6 and HHV-7: 2 cases, HHV-6 and EBV: 2 cases, HHV-7 and EBV: 2 cases, EBV and bacterial 16S: 1 case), and even triple infection (HHV-6, HHV-7, EBV: 1 case) were detected in the tumor specimens from 8 of 22 patients in IgG4-ROD. In addition, quadruple infection (HHV-6, HHV-7, EBV, and bacterial 16S) was detected in only one patient with orbital RLH among all patients studied.

## Discussion

Demonstrating the presence of pathogens in patients with lymphoma tissues is challenging. To our knowledge, this is the first multiple virus detection in LPD samples performed using multiplex and quantitative real-time PCR assays that allow simultaneous determination of various genomic DNA pathogens. In addition, our study is the first to report the presence of HHV-6, HHV-7, and EBV in orbital inflammatory tumors such as IgG4-ROD and orbital RLH, demonstrating an association between viral antigen expression and LPDs. This result supports the hypothesis that certain infections, particularly those caused by HHV-6, HHV-7, and EBV, are related to specific cases of malignant and benign LPDs of the ocular adnexa.

Malignant tumors typically linked to EBV include Burkitt’s lymphoma nasopharyngeal carcinoma, T-cell lymphoma, and Hodgkin’s lymphoma. Evidence for the oncogenic potential of EBV derives from its ability to infect and transform normal human B cells *in vitro*, manipulating differentiation signaling pathways and leading to continuously growing lymphoblast cell lines and potentially immortal cell lines^13^. This germ cell expansion can lead to oncogenic rearrangements such as a t(8:14) c-myc translocation and result in the development of malignant lymphoma^14^,^15^,^16^. Additionally, the presence of EBV has a significant effect on the clinical outcome of lymphomas, including diffuse large B-cell lymphoma. These observations raise a question of whether the presence of EBV in ocular LPD can be predictive of clinical outcome^13^. Our study demonstrated an even stronger association between EBV and IgG4-ROD or RLH compared to conjunctival MALT lymphoma (31.8% or 28.6% versus 15.8%), suggesting that the malignant potential of EBV occurs in conjunction with a primarily increased risk of the development of benign LPDs of the ocular adnexa. One investigation of the association of EBV and benign LPDs identified a 58% rate of EBV reactivation in patients with IgG4-ROD and an 18% rate of reactivation in patients with RLH^3^. Our study also demonstrated a similar association between HHV-6 and HHV-7 viruses of the herpes family and LPDs.

HHV-6 and HHV-7 are established oncogenic viruses that can induce tumors in an animal model through the inactivation of p53 and deregulation of nuclear factor kappa-B^17^,^18^. Known malignant tumors typically linked with HHV-6 include T-cell lymphoma and Hodgkin’s lymphoma. Although HHV-6 displays tropism for T cells, the prevalence of HHV-6 DNA in biopsied specimens with B-cell lymphoma or Hodgkin’s disease is 22.2% or 35.1%, respectively^19^. Reactivation of HHV-6 is also observed in gastrointestinal polyps, with one group reporting HHV-6 antigen expression in biopsy specimens from 62.5% of patients with gastric adenomas and 87.5% of patients with tubulovillous adenoma, with no detection in mucosal samples from healthy controls^20^. Although the incidence of HHV-6 detection in conjunctival and orbital MALT lymphoma was lower (5.2% and 6.7%, respectively) in the present study, its association with IgG4-ROD was more robust. In cases of IgG4-ROD, 5 of 22 cases (23%) were positive for HHV-6 DNA, ranging from 8.1 × 10^2^ to 6.7 × 10^4^ copies (mean, 1.7 × 10^3^ copies/μg DNA). The number of RLH patients was relatively low with 7 samples studied; however, 3 of these samples (43.9%) contained detectable HHV-6, establishing a correlation that warrants further evaluation. HHV-7 DNA is closely related to HHV-6 and establishes a life-long infection following exposure, primarily within the salivary glands and lymphoreticular system, similar to HHV-6. Our study revealed the HHV-7 genome in 8 of 22 IgG4-ROD patients studied (36.4%), 3 of 7 RLH samples, and 1 of 19 MALT lymphoma samples. These results taken together indicate that HHV-6 and HHV-7 have a stronger relationship with benign orbital LPDs than MALT lymphomas. Further studies are necessary to examine whether HHV-6 and HHV-7 DNA are also related to other malignant LPDs such as Mantle and T-cell lymphoma, as well as the pathophysiology of their association with LPDs.

If an association between infectious agents and LPDs exists, it is unclear how the infectious agents induce these LPDs of the ocular adnexa. One possibility is that infectious antigens expedite or are required for the expansion of the monoclonal/oligoclonal B cells that are found in either benign or malignant LPDs during the germinal center reaction. As HHV-6, HHV-7, and EBV continuously infect the salivary glands^21^, it is conceivable that they may also continuously infect lacrimal glands and conjunctiva. Through local and chronic stimulation, these continuous infections as exogenous or endogenous antigens may trigger infectious oncogenes. Further studies are needed to examine whether these infectious antigens consequently induce the proliferation of lymphocytes and whether they play a primary or secondary pathogenic role.

Moreover, although continuous infections of lymphocytes by HHV-6, HHV-7, and EBV were reported in this present case, pathogenic DNA was not detected in peripheral blood (data not shown), suggesting local infection of the tumor. Taking into account the prevalence rate for these viruses in the salivary glands of healthy individuals, we can postulate that the interaction between these viral infections and other environmental risk factors increases the risk for developing LPDs in conjunction with genetic risk factors (related to immune/infectious responses) through undetermined underlying mechanisms.

Mechanisms consistent with the pathogenesis of MALT lymphoma, such as the expression of viral oncogenes leading to the stimulation of a Th2 immune response, may play a role in the polyclonal or monoclonal proliferation of LPDs^22^. The predominantly Th2 immune response and increased production of Th2-type cytokines, such as interleukin (IL)-4, IL-5, IL-10, and IL-13, have also been reported in patients with IgG4-ROD^23^,^24^; but, the source of the putative antigen-reacting Th2 cells (CD4^+^ lymphocytes) involved in this process remains largely unknown. Virus-induced IL-10 plays an important role in Th2 and regulatory T cells, acts as a potent factor of B cell survival, and is produced after viral stimulation^25^. Although many investigators reported that IgG4 disease is Th2 dominant, the identity of the antigen that these Th2 cells are responding to is unclear. Thus, it is possible that these detected viruses may activate the Th2 cells that cause the pathology of IgG4, RLH, and MALT lymphoma. In fact, HHV-6 and HHV-7 can infect latently CD4^+^ T cells, and they proliferate in the cells.

Infectious agents were more common in patients with conjunctival MALT lymphoma than in those with orbital MALT lymphoma, indicating that there may be different pathways leading to lymphomagenesis. This observation is similar to what we reported after using high-resolution genomic copy number profiling by single nucleotide polymorphism microarrays^26^. These infectious agent-driven polyclonal B-cell proliferations could be an initial molecular event or process that could lead to or increase the probability of a real clonal expansion over time. If these infections play an etiologic role in benign and malignant LPDs, the elimination of these infectious agents might facilitate control of these diseases. Double and triple infections were detected in some of the patients with LPDs of the ocular adnexa. However, the clinical relevance of multiple infections is currently unclear, and warrants further studies, which may lead to a novel method to reduce the probability of benign and malignant lymphadenopathy. For example, considering that HHV-6 is the cause of exanthema subitum, treatment for HHV-6, such as ganciclovir, may also be useful for treating IgG4-related diseases by suppressing viral RNA expression. Similarly, other antiviral therapies might be used as prophylaxis or treatment for patients at higher risk for developing LPDs from these ubiquitous pathogens.

For a large part of the population, these infections are acquired at a young age and remain latent in the body for life^27^,^28^. It is speculated that most individuals are latently infected by these viruses at levels that are insufficient to determine viral load in peripheral blood mononuclear cells until they become reactivated, such as in case of immunosuppression. This concept is consistent with previous reports indicating that molecular detection of these viruses by PCR is not always associated with the presence of lymphocytic infiltrates in tissue^29^. Because these viruses are latent in lymphocytes, false-positive PCR detection in the biopsy specimens could have occurred. Furthermore, symptomatic viral reactivations are associated with high levels of viral loads (>500 copies/μg of DNA), while values of <100 copies/μg of DNA are probably indicative of latent virus infections^30^. Our analysis, however, could not directly distinguish whether these viruses persist in a latent or reactivated state. Because of the nature of biopsy sampling, there is a high level of difficulty in obtaining lacrimal tissue from healthy individuals. This lack of control tissue prevented us from directly comparing the incidence of virus detection between patients with LPD and healthy controls and from establishing a baseline of latent viral expression. Therefore, it is possible that the detection of viral antigens may be related to a latent or non-contributory infection, potentially of the infiltrating inflammatory cells rather than the adnexal tumor itself, whose presence may be expected in a common infection acquired early in life. Our PCR system is less likely to detect positive latent samples, however, than a qualitative PCR assay, which picks up more latent viruses. By using the multiplex PCR system as an initial screening followed by an additional quantitative PCR analysis, we reduced the probability of latent or non-contributory infections being detected and reported^31^. Finally, although our protocol allowed us to evaluate the presence of the majority of known oncogenic pathogens, our PCR system does not include every potentially oncogenic pathogen. Some notable examples worth evaluating in the future for completeness includes the human papilloma virus family, parvovirus, hepatitis B and C viruses, Merkel cell polyomavirus, and simian virus 40.

In summary, HHV-6, HHV-7, and EBV may be associated with the pathogenesis of benign and malignant LPDs of the ocular adnexa. The exact mechanism of the role of these viruses, and thus the clinical significance, is not yet clear. Presence of DNA of the infectious agents could also be co-incidental/epithenomenon. This possibility is unlikely, as normal conjunctival and orbital tissues did not reveal the presence of DNA of agents. These findings, however, open a new perspective for further research of LPDs of the ocular adnexa. Further efforts are needed to elucidate the relevance of the molecular detection of these viruses in the context of LPDs of the ocular adnexa. Additionally, further studies of the mechanisms underlying malignant and benign LPDs are needed to clarify the potentially inductive roles of environmental and pathogenic stimuli.

## Methods

### Patients, Sample Collection, and Diagnosis

We studied 70 patients (32 men and 38 women) diagnosed with LPDs of the ocular adnexa, including 19 conjunctival MALT lymphomas (7 men and 12 women, mean age 50.8 ± 18.0 years), 15 orbital MALT lymphomas (7 men and 8 women, mean age 70.8 ± 13.9 years), 7 orbital difuse large B-cell lymphomas (DLBCL; 4 men and 3 women, mean age 64.4 ± 14.2 years), 22 IgG4-ROD (12 men and 10 women, mean age 60.5 ± 15.5 years), and 7 orbital RLH (2 men and 5 women, mean age 55.7 ± 13.4 years) from 2008 to 2013 at the Department of Ophthalmology, Tokyo Medical University. This study was approved by Tokyo Medical University institutional review board. All experimental methods were conducted in accordance with the Declaration of Helsinki. All patients were Asian and immunocompetent adults. Surgically resected or biopsied specimens of LPDs of the ocular adnexa were delivered immediately to the Pathology and Molecular Laboratory. All patients gave informed consent to the collection and analysis of samples.

The diagnosis of each LPD of the ocular adnexa was established on the basis of clinical, radiographic, histologic, and molecular genetic analyses such as gene rearrangement. Specifically, the diagnosis of IgG4-ROD was made in accordance with recently published criteria^5^,^32^. Conventional histologic and immunohistochemical evaluations were performed on resected tissues fixed in 10% formaldehyde to evaluate features of the LPDs. B-cell monoclonal expansion was confirmed by flow cytometry analysis and immunoglobulin heavy chain (IgH) gene rearrangements using Southern blot analysis or PCR for conjunctival MALT lymphoma (SRL, Tokyo, Japan). IgG4 values higher than the normal range of 4.8–105 mg/dl were used as diagnostic criteria to establish the presence of IgG4-ROD. Patient characteristics and presence of monoclonality are summarized in Table 3.

### Polymerase Chain Reaction Analyses

Specimens were screened for bacterial, viral, fungal, and parasitic DNA by multiplex and real-time PCR assays, and the quantity of microorganism DNA was measured using broad-range real-time PCR, as described previously^12^. Briefly, DNA was extracted from the tumor specimens using a DNA Mini Kit (Qiagen, Valencia, CA). Genomic DNA of eight human herpes viruses (HHV1–8), toxoplasma, bacteria, and fungi in the tumor specimens was measured by two independent PCR assays: (*1*) multiplex and real-time PCR and (*2*) broad-range real-time PCR.

The multiplex PCR was designed to qualitatively measure genomic DNA of 8 HHVs (HSV-1, HSV-2, varicella-zoster virus (VZV), EBV, cytomegalovirus (CMV), HHV-6, HHV-7, and HHV-8), BK virus, JC virus, human T-lymphotrophic virus (HTLV-1), Coxsackie virus, enterovirus, influenza virus, *Bartonella*, chlamydia 23S, *Acanthamoeba*, toxocara, and toxoplasma. If the multiplex PCR results were positive, real-time PCR was then performed. The broad-range real-time PCR was performed to detect the genome for bacterial 16S rDNA or fungal 18S/28S rDNA^33^. PCR was performed with a LightCycler 480 II instrument (Roche, Basel, Switzerland). Real-time PCR was performed using the AmpliTaq Gold and the Real-Time PCR 7300 system (Applied Biosystems, Foster City, CA) or LightCycler 480 II. Primers and probes for each pathogen and the PCR conditions were described previously^33^,^34^,^35^,^36^. Amplification of the human *gamma*-globulin gene served as an internal positive extraction and amplification control. Copy number values of more than 50 copies/μg DNA (virus and parasite) or 100 copies/μg DNA (bacteria and fungi) in the biopsy sample were considered to be significant^34^.

## Additional Information

**How to cite this article**: Usui, Y. *et al.* Comprehensive polymerase chain reaction assay for detection of pathogenic DNA in lymphoproliferative disorders of the ocular adnexa. *Sci. Rep.* **6**, 36621; doi: 10.1038/srep36621 (2016).

**Publisher’s note:** Springer Nature remains neutral with regard to jurisdictional claims in published maps and institutional affiliations.

## Figures and Tables

**Table 1:** Positive rates for various microorganisms detected by comprehensive polymerase chain reaction assays in tumor specimens of 70 patients with lymphoproliferative disorders of the ocular adnexa.

Infectious agent	Multiplex PCR and Real-time PCR				
	Conjunctival MALT	Orbital MALT	Orbital DLBCL	IgG4-ROD	Orbital RLH
EBV	3/19 (15.8%)	0/15 (0%)	2/7 (28.6%)	7/22 (31.8%)	2/7 (28.6%)
HHV-6	1/19 (5.2%)	1/15(6.7%)	0/7 (0%)	5/22 (22.7%)	2/7 (28.6%)
HHV-7	1/19 (5.2%)	0/15 (0%)	0/7 (0%)	8/22 (36.4%)	3/7 (42.9%)
Chlamydia	1/19 (5.2%)	0/15 (0%)	0/7 (0%)	0/22 (0%)	0/7 (0%)
Bacteria	2/19 (10.5%)	0/15 (0%)	1/7 (14.3%)	1/22 (4.5%)	1/7 (14.3%)

PCR, polymerase chain reaction; MALT, mucosa-associated lymphoid tissue lymphoma; DLBCL, diffuse large B-cell lymphoma; IgG4-ROD, IgG4-related ophthalmic disease; RLH, reactive lymphoid hyperplasia; EBV, Epstein-Barr virus; HHV, human herpes virus.

**Table 2:** Multiple positive rates detected by comprehensive polymerase chain reaction assays in biopsy specimens of 70 patients with lymphoproliferative disorders of the ocular adnexa.

	Number of patients with double positive (%)	Number of patients with triple positive (%)	Number of patients with quadruple positive (%)
Conjunctival MALT	2/19 (10.5)	0/19 (0)	0/19 (0)
	HHV-6 + EBV: 1 case		
	HHV-7 + EBV: 1 case		
Orbital MALT	0/15 (0)	0/15 (0)	0/15 (0)
Orbital DLBCL	1/7 (14.3)	0/7 (0)	0/7 (0)
	EBV + Bacteria: 1 case		
IgG4-ROD	7/22 (31.8)	1/22 (4.5)	0/22 (0)
	HHV-6 + HHV-7: 2 cases	HHV-6 + HHV-7 + EBV: 1 case	
	HHV-6 + EBV: 2 cases		
	HHV-7 + EBV: 2 cases		
	EBV + Bacteria: 1 case		
RLH	0/7 (0)	0/7 (0)	1/7 (14.3)
			HHV-6 + HHV-7 + EBV + Bacteria: 1 case

MALT, mucosa-associated lymphoid tissue lymphoma; DLBCL, diffuse large B-cell lymphoma; IgG4-ROD, IgG4-related ophthalmic disease; RLH, reactive lymphoid hyperplasia; HHV, human herpes virus; EBV, Epstein-Barr virus.

**Table 3:** Patient characteristics and monoclonality status of IgH gene rearrangement.

	Conjunctival MALT	Orbital MALT	Orbital DLBCL	IgG4-ROD	Orbital RLH
Number	19	15	7	22	7
Age (yrs)	50.8 ± 18.0	70.8 ± 13.9	64.4 ± 14.2	60.5 ± 15.5	55.7 ± 13.4
Men/Women	7/12	7/8	4/3	12/10	2/5
IgH gene rearrangement	all (+)	all (+)	all (+)	all (−)	all (−)

MALT, mucosa-associated lymphoid tissue lymphoma; DLBCL, diffuse large B-cell lymphoma; IgG4-ROD, IgG4-related ophthalmic disease; RLH, reactive lymphoid hyperplasia; IgH, immunoglobulin heavy chain.
